# Monitoring of an electrically induced signal in melon in relation to different environmental conditions in a smart farm

**DOI:** 10.1186/s13765-022-00749-z

**Published:** 2022-12-09

**Authors:** Jin Hee Park, Gyung Min Park, Eun Jeong Kim, Yu Min Jeon

**Affiliations:** 1 Department of Environmental and Biological Chemistry, Chungbuk National University, Cheongju, 28644 Republic of Korea; 2 Watermelon and Strawberry Research Institute, Division of Research and Development, Chungcheongbuk-Do Agricultural Research and Extension Services, Eumsung, 27668 Republic of Korea

**Keywords:** Plant induced electrical signal, Smart farm, Melon, Growth, Environmental condition

## Abstract

A smart farm that automatically regulates environmental conditions such as temperature, humidity and nutrient supply will maximize crop production per unit area by using ICT-based technology. To control the environment in a smart greenhouse, plant growth should be monitored in real time. The physiological activity of a plant was monitored by receiving an electrical signal from inside the plant stem which changed when the plant absorbed nutrients and water. In this study, the environmental conditions in different areas of a smart farm were not much different, and growth parameters were not much affected by the environmental conditions of the area. However, a plant induced electrical signal (PIES) was associated with the atmospheric and media temperature and relative humidity although there was time lag of 6 ~ 7 h for the peaks of the PIES and other environmental conditions. Therefore, monitoring a PIES will make it possible to improve the growth environments in smart farms.

## Introduction

The proportion of the global population malnourished due to hunger showed a trend of decreasing from 13.30 to 8.10% from 2002 to 2019 but increased to 9.2 ~ 10.4% after 2020 when the coronavirus pandemic began [11]. Because of an increase in population growth and climate change affecting food production, efforts to increase sustainable food production are required [14]. Declining agricultural populations and agricultural land area can also affect food security and supply. Between 2012 and 2020, the ratio of the domestic farm household population to agricultural land area continued to decrease [16]. The decrease in farmland area and the aging of the farm household population in Korea will require efforts to increase productivity per unit area with less manpower. Therefore, new agricultural technologies will have to be developed to solve the food problem in the future.

To increase production per farmland area and labor, various methods such as hydroponics, plant factories, and container farms with high-density indoor cultivation have been introduced, among which smart greenhouses are a key solution for the optimization of environmental conditions and precision agriculture [3, 13]. Precision agriculture is a system that optimizes production management by collecting information on factors affecting crop cultivation based on information and communications technology (ICT) and minimizing unnecessary agricultural materials [12]. ICT emphasizes long-distance communication including storage, transmission, and manipulation of data by accessing information such as big data, the cloud, and artificial intelligence [28]. Therefore, the collection of various growth-related data of plants is required for precision agriculture.

A smart greenhouse has technology that collects growth and environmental data using ICT, diagnoses the environmental conditions and adjusts the condition using devices such as fans, heaters, and air conditioners suitable for plant growth by sensor monitoring [31]. Therefore, in a smart greenhouse, the manpower can be minimized while the temperature, humidity and supply of nutrients are automatically controlled to an optimal state for plant growth. However, the aging of farm population has difficulty understanding ICT and its equipment, which is the core of precision agriculture. In addition, expensive equipment raises the entry barrier for smart greenhouses [18, 32]. Therefore, decision making on environmental conditions also needs to be controlled automatically without human intervention based on the monitoring of plant bioinformatics.

Methods used to evaluate plant activity during plant growth include measurements of photosynthesis, chlorophyll content and reactive oxygen species, which are destructive and cannot be continuously monitored [8, 17]. To evaluate plant growth in a non-destructive and real-time manner, a plant induced electrical signal (PIES) can be used [23]. Xylem sap flow is also non-destructively measured to understand plant reaction in relation to environmental variables [4]. However, sap flow is only related to water movement and heat should be applied. Because the sap flow measurement was done by the heat balance or dissipation of the moving sap, plant stem can be damaged by the heat and continuous measurement of sap flow is restricted [4, 23]. However, PIES measurement does not introduce heat and reflect both water and nutrient uptake by the plant [23]. Because the PIES is related to the movement of water and nutrients in plant stems indicating the plant physiological activity, monitoring of the PIES can be used to control and optimize the environmental conditions in smart farms. Many researches on smart farm system focused on monitoring and controlling environmental parameters such as temperature, humidity, soil water content and pH to enhance productivity and efficiency in agriculture [19]. However, environmental parameters do not directly reflect plant physiological conditions and precise management of optimum conditions for plant growth can be achieved by monitoring of plant physiological response. Because the PIES measures electrical conductivity inside plant stem which is related to plant physiological activity, smart farm systems such as irrigation, ventilation, heating, cooling and shading can be controlled based on the PIES.

In our previous study, PIES of paprika was shown to be positively correlated with irradiation and temperature and negatively correlated with relative humidity [23]. However, PIES of broccoli was not promptly affected by changes in light and CO_2_ concentrations because water and nutrient transport was not changed immediately [22]. Under different urea fertilization, the PIES was related to nitrate content in broccoli stem [15]. Although previous research showed that the PIES can be used to monitor plant physiological responses under different environmental conditions, it was monitored for some limited plants including paprika, pepper and broccoli. Therefore, to use PIESs in smart farms, first the PIES data for various plants under various environmental conditions in a smart farm should be collected and interpreted. Melons are an important horticultural crop worldwide and are characterized by high yields in environments that are hot and dry [2, 29]. Especially, Cucurbitaceae including melon and watermelon are sensitive to relative humidity and temperature and melons require sufficient light intensity, which affect productivity [5]. Therefore, the objective of this study was to evaluate the PIES of melon and its relevance to environmental and growth factors for possible application to monitoring of bioinformatics in controlling environmental conditions in smart farms.

## Materials and methods

### Growth conditions of the melons in the smart farm

The experiment was conducted at a greenhouse (330 m^2^) in Republic of Korea, and the variety of melon (*Cucumis melo* L.) used was D’Artagnan. Melon seedlings were transplanted at 30 cm spacing on the 5th of July, and the melons were harvested on the 15th of September 2021. As for the growth conditions, the indoor temperature was maintained at 28 ~ 33 ℃ during the day and 18 ~ 22 ℃ at night using a smart environmental controller (Farm morning, Green labs, Korea) [25]. The melons were grown in coir slab (chip:dust ratio of 7:3). Stock nutrient solution contained 60.6 g/L of KNO_3_, 32.6 g/L of Ca(NO_3_)_2_·4H_2_O, 2 g/L of Fe-EDTA, 15.2 g/L of NH_4_H_2_PO_4_, and 36.9 g/L of MgSO_4_·7H_2_O and diluted solution was supplied. Nutrients were supplied based on EC through the nutrient phase (Farm morning, Green labs, Korea), which were 1.5, 1.8, 2.0 and 2.3 dS/m for each growth stage of transplanting, 14 days after transplanting, fruiting and 20 days after fruiting, respectively. Irrigation was controlled to 30% in the early growth period, 20% after the fruiting stage, and 10% at 10 days before harvest based on the amount of drainage. Pesticide was applied three times to control greenhouse whitefly*.*

### Monitoring of environmental conditions in the smart farm

Experimental areas were divided into 3 different areas to evaluate effect of slightly different environmental conditions on plant growth in smart farm. Size of each area was 41.25 m^2^. In each area, the environmental conditions were monitored using one set of environmental sensors including atmospheric temperature, relative humidity, CO_2_, photosynthetic photon flux density (PPFD), media electrical conductivity (EC), media temperature, and media moisture. As environmental sensors, SH-VT260 (SOHA Tech) was used for the air temperature, relative humidity, and CO_2_ sensors; TEROS 12 (METER Group) was used for the media temperature, media moisture, and EC. PAR photon flux sensor (Apogee instruments) was installed for monitoring the light intensity. Environmental data were measured every minute and averaged in units of 1 h. Although the environmental data were collected during the growth of the melons, environmental data for some selected period was presented in Figures to match the data with PIES data and growth parameters measured.

### Monitoring of the plant induced electrical signal in the melons

Two electrodes with three needles made of stainless steel were inserted into the stem of a melon about 10 cm from the media surface. The electrode was connected to a Juns meter OL (Purumbio), and the electrical resistance was measured and converted into the electrical conductivity of the stem. The electrical conductivity of the plant was denoted as the plant induced electrical signal (PIES) to avoid confusion with soil EC. Three to four plants in each area were monitored for PIES and growth parameters of the plants were investigated. The PIES data were measured every 10 min from August to September and averaged in units of 1 h.

### Melon growth investigation

The growth and quality of the melons were investigated in accordance with the manual for agricultural investigation of the Agricultural Science and Technology Research [25]. The growth of the melons was investigated by measuring the stem length, number of leaves, leaf length, leaf width, stem diameter, number of flowers, fruit weight, fruit circumference, fruit length, thickness of pericarp, sugar content, and net formation index (1, excellent; 2, good; 3, average; 4, poor; 5, bad) every 3 ~ 4 days.

### Statistical analysis of the data

Statistical analysis of the data was performed using the SPSS software (IBM, USA). The differences among the different areas were examined by one-way analysis of variance (ANOVA) followed by Duncan’s multiple range test. Principal component analysis (PCA) was performed using XLSTAT.

## Results

### Monitoring of environmental conditions

**Fig. 1:**
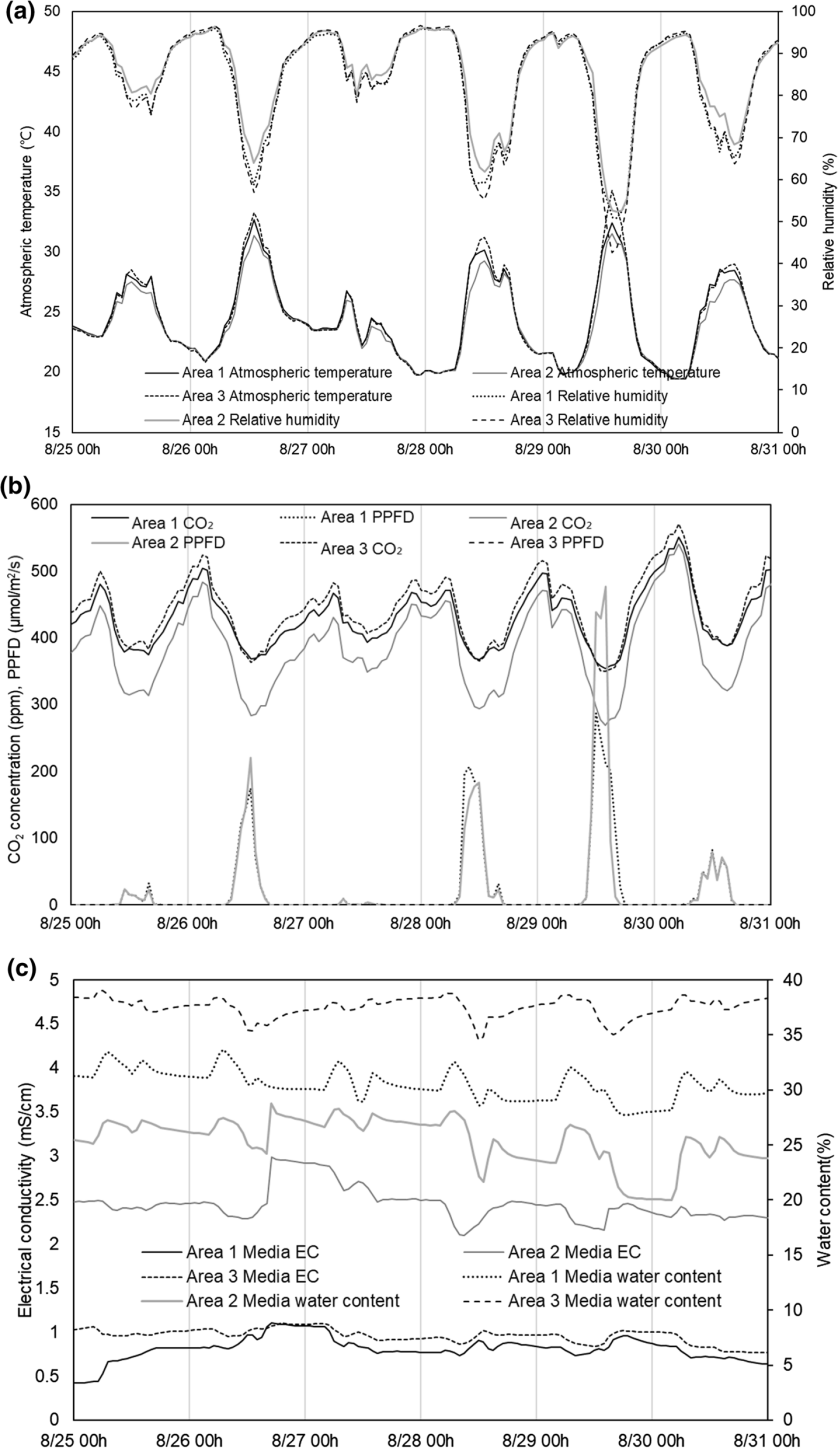
Environmental conditions (**a** atmospheric temperature, relative humidity, **b** CO_2_ concentration, photosynthetic photon flux density (PPFD), **c** electrical conductivity and water content of media) monitored in a melon smart farm

The environmental conditions of the different areas in the smart farm were slightly different during the daytime, but the difference was not found during the nighttime. The temperature was the highest in Area 3 and the lowest in Area 2 while the relative humidity was inversely related to temperature and showed the lowest relative humidity in Area 3 (Fig. 1a). The temperature reached its peak after 1 h of the peak PPFD, and the CO_2_ was the highest when the PPFD reached the highest level indicating that CO_2_ assimilation was related to the PPFD [22]. The PPFD of Area 3 was missing because of a sensor failure. The PPFD of Area 2 was higher than that of Area 1, and the CO_2_ concentration was lower in Area 2 (Fig. 1b). However, the lower CO_2_ concentration in Area 2 than the other areas might be attributed to the position of the area located in the middle of the smart farm. During the experimental period, the melons were irrigated from 5:30 ~ 7:40 am and 1:30 ~ 2:40 pm, and the water content increased when the media was irrigated. The water content and EC were different between the three areas, which might be related to different nutrient and water uptake by the plants in the different slabs. The increase in water content was attributed to the irrigation that decreased the media EC (Fig. 1c). The media temperature showed a similar trend to the atmospheric temperature but reached its peak after about 1 h.

### Environmental factors affecting the PIES

**Fig. 2:**
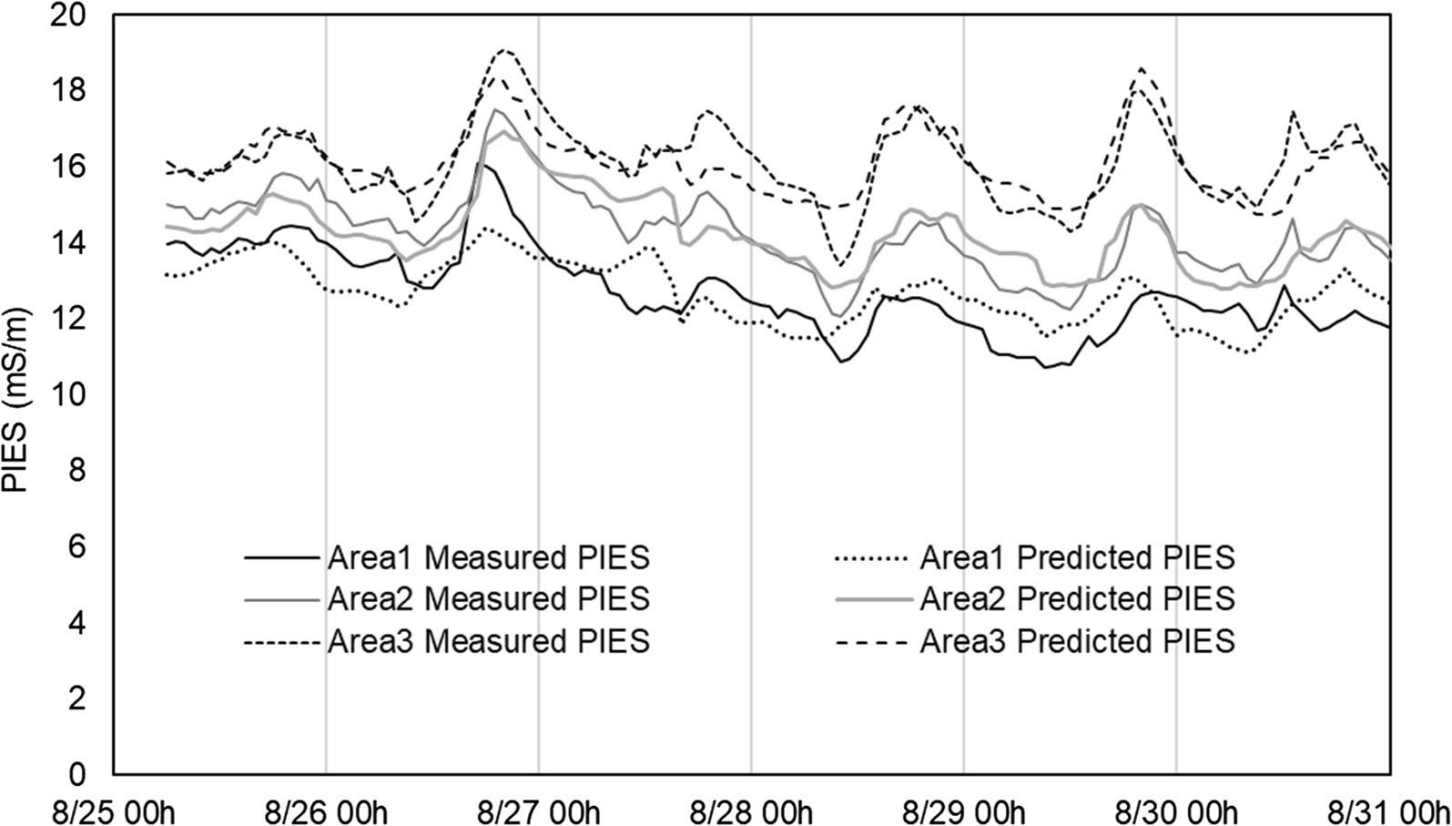
Plant induced electrical signal (PIES) of melons grown in different areas of a smart farm

The magnitude of the PIES in the different areas was in the order of Area 3 > Area 2 > Area 1 (Fig. 2). Although the PIES showed a diurnal variation with the PPFD and atmospheric temperature, the PIES increased 7 h after the temperature increased indicating that transpiration was retarded. Although there was a time lag between the PIES and environmental conditions, the diurnal pattern of the PIES was similar to the atmospheric temperature, media temperature, and irradiation and showed an opposite pattern to the relative humidity and carbon dioxide. Because the peak time of the PIES occurred 6 ~ 7 h after the peak of the atmospheric temperature, relative humidity, CO_2_ concentration and PPFD and 5 ~ 6 h after the peak of the media temperature and water content and 2 h after the peak of the media EC, the environmental data were moved back, and stepwise regression was conducted to investigate the environmental factors affecting the PIES.

According to the stepwise regression, the atmospheric and media temperature was the main driving factor for the PIES variation, and the media EC, water content and relative humidity were also selected as predictors (Table 1). Because the environmental factors were related to the PIES, the PIES could be predicted using the result of the stepwise regression. The predicted PIES was well fitted to the monitored PIES with a coefficient of determination ranging from 0.50 to 0.76 (Fig. 2). The lowest coefficient of determination was found in Area 1, where other factors might affect the PIES in addition to the environmental conditions.

**Table 1:** Results of the stepwise regression for the PIES

Samples	Variables	Beta standardized coefficient	Adjusted R^2^
Area 1	Media temperature	0.491**	0.489
	Media water content	0.179*	
	Relative humidity	0.715*	
	Atmospheric temperature	0.692	
Area 2	Media EC	0.407**	0.751
	Atmospheric temperature	0.883**	
	Relative humidity	0.428**	
	Media water content	0.129*	
Area 3	Atmospheric temperature	0.743**	0.667
	Media EC	0.196**	

^*^p < 0.05. **p < 0.01

### Growth parameters of the melons and their relation to the PIES

Plant growth parameters such as shoot length, number of leaves, leaf length and width, and stem diameter were measured on the 24th, 27th and 31st of August and 3rd of September, which were averaged and used for PCA. The characteristics of the fruit such as the fruit weight, circumference, length, width, thickness of the pericarp, sugar content and net formation were analyzed on the day of the harvest. The growth parameters were not much different among the melons grown in different areas because the environmental factors were not different enough to affect the melon plant growth. Some fruit characteristics such as fruit circumference and width were significantly lower in Area 1, but other factors were not much different (Table 2). Therefore, the overall plant growth and fruit quality were not affected by the slightly different environmental conditions.

**Table 2:** Growth parameters and yield components of the melons grown in different areas in the smart farm

	Area1	Area2	Area3
Shoot length (cm)	165.23 ± 12.76 a	169.80 ± 33.87 a	134.13 ± 29.72 a
Number of leaves	36.33 ± 2.89 a	39.33 ± 5.03 a	33.50 ± 3.32 a
Leaf length (cm)	12.39 ± 0.77 a	13.03 ± 1.67 a	12.43 ± 0.41 a
Leaf width (cm)	19.81 ± 5.62 a	16.86 ± 1.87 a	16.14 ± 1.66 a
Stem diameter (cm)	11.64 ± 0.69 a	11.74 ± 0.73 a	12.11 ± 0.70 a
Fruit weight (g)	1766.67 ± 106.93 a	1876.67 ± 261.02 a	1675.00 ± 49.33 a
Fruit circumference (cm)	48.70 ± 1.13 b	51.53 ± 2.41 a	49.40 ± 0.33 ab
Fruit length (cm)	14.30 ± 1.01 a	14.27 ± 0.97 a	14.28 ± 0.15 a
Fruit width (cm)	14.77 ± 0.25 b	16.05 ± 0.21 a	15.10 ± 0.24 b
Pericarp (cm)	0.12 ± 0.03 a	0.13 ± 0.06 a	0.10 ± 0.00 a
Sugar content (°Bx)	9.83 ± 1.26 a	10.33 ± 0.58 a	9.88 ± 0.63 a
Net formation index	3.00 ± 0.00 a	3.67 ± 2.31 a	3.50 ± 1.00 a

Difference letters in the same row indicate significant differences according to Duncan’s multiple range test (p<0.05)

**Fig. 3:**
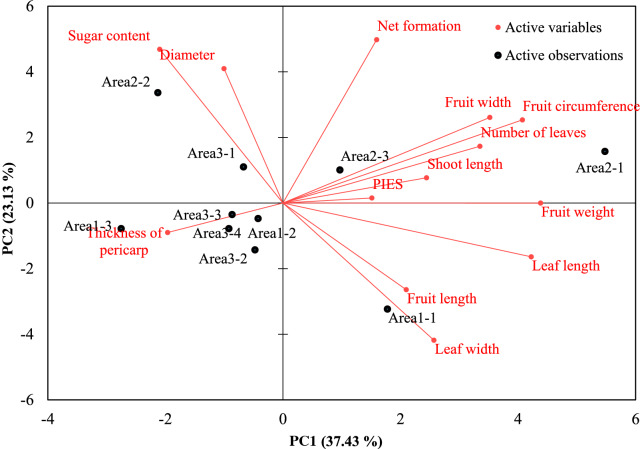
Biplot of the principal component analysis for the growth parameters of melons grown in different areas of a smart farm

PCA was performed with an individual dataset of 10 different plants from three different areas, and the projections of the cases with two principal components were imaged. PC1 and PC2 accounted for 37.4% and 23.1% of the total variation, respectively. Although the melons from different areas did not show a clear separation, the melons from Area 2 were separated from the melons from Areas 1 and 3 (Table 3 and Fig. 3). PC1 was strongly associated with the plant and fruit growth parameters such as shoot length, number of leaves, leaf length and fruit weight while PC2 was related to fruit quality. However, the PIES was not significantly related to the growth parameters (Table 3 and Fig. 3).

**Table 3:** Principal component loadings and variance explained by the first four principal components

	PC 1	PC 2	PC 3	PC 4
PIES	0.321	0.026	0.423	− 0.800
Shoot length	**0.518**	0.128	0.381	**0.728**
Number of leaves	**0.710**	0.288	0.357	0.284
Leaf length	**0.895**	− 0.273	− 0.040	− 0.191
Leaf width	**0.544**	− 0.696	0.066	0.176
Diameter	− 0.212	**0.683**	**0.605**	− 0.015
Fruit weight	**0.929**	0.000	0.113	0.104
Fruit circumference	**0.864**	0.422	− 0.211	− 0.032
Fruit length	0.445	− 0.440	**0.610**	− 0.266
Fruit width	**0.746**	0.435	− 0.391	− 0.149
Thickness of pericarp	− 0.416	− 0.150	**0.568**	0.010
Sugar content	− 0.444	**0.780**	0.201	0.052
Net formation	0.338	**0.829**	− 0.103	− 0.184
Eigenvalue	4.866	3.007	1.778	1.460
Variability (%)	37.434	23.127	13.679	11.234
Cumulative %	37.434	60.561	74.240	85.475

The bold value indicates a loading >0.5

## Discussion

The PIES was related to the water and nutrient uptake of the plants because it measured the electrical resistance between the electrodes inserted in the stem which was converted to electrical conductivity [23]. Generally, water and nutrient uptake by plants is related to environmental conditions. When paprika was grown in February and May and monitored, the PIES increased with the increasing atmospheric temperature [23]. However, in this study, there was time lag of 7 h between the PIES and atmospheric temperature increase, which might be attributed to retarded transpiration due to high temperature during the daytime. Xin et al. [33] also reported 1 ~ 3 h time lag between the sap flow of the Common Camellia and the illumination intensity or air temperature. High temperature induces stomatal closure leading to a declined sap flow in Eucalyptus species [9]. Stomatal conductance of banana leaves was the highest before noon, but transpiration reached its peak between 1 and 4 pm, but in summer, the peak of transpiration occurred at 11 am [26]. Therefore, the diurnal variation of a PIES according to the temperature or irradiation can be seasonally different because of high temperature stress in the summer.

Stepwise regression analysis provides information on the environmental conditions affecting the PIES [6]. Although selected parameters for stepwise regression was not consistent in the different areas, some common factors were temperature related factors such as atmospheric and media temperature. Root zone temperature influences the physiological response including ion uptake, root growth, chemical reactions and water and nutrient transport in soil [24]. Relative humidity was also selected as a parameter in the model of Areas 1 and 2. Vu et al. [30] reported that water uptake by rice was influenced by a vapor pressure deficit, but the root zone temperature had no effect. PPFD was not included in the model because it was zero during nighttime, but it was positively related to atmospheric temperature during daytime and indirectly affected PIES because atmospheric temperature began to increase with increase of PPFD [21]. The CO_2_ was not included because it decreased as the result of the increased PIES rather than the cause of PIES increase. Photosynthesis resulted in CO_2_ decrease of 7 ~ 10 μmol/mol compared to ambient CO_2_ baseline during the daytime [7].

Because the physiological response is associated with environmental conditions, abiotic stresses such as salinity and heat lead to abnormal behavior of plants due to physiological damages [10]. Abiotic and biotic stress induced changes in nutrient uptake, which is important factor in diagnosing abnormal metabolism and reduced yield of plant [1]. When a plant was exposed to stress conditions, the PIES did not respond to environmental conditions and could not be predicted by the environmental conditions [22, 23]. In this case, a comparison of the monitored PIES and predicted PIES will help in diagnosing plant stress in smart farms and improving growth conditions for plants in their early stages of stress [23].

PCA is used to find variables associated and relationships among samples and to reduce the dimension [27]. PCA was conducted to evaluate the relationship between the PIES and growth parameters. Although the PIES was not significantly related to the growth parameters of the melons, it was nearly located in the same plane with plant and fruit growth parameters. Environmental conditions such as temperature and PPFD were not much different in each area of the smart farm, which did not affect the plant development, growth and biomass. The difference in plant growth was due to the individual plants rather than the differences in the environmental conditions. In addition, other environmental aspects such as water and nutrient supply would also be critical in the interpretation of plant responses and thus, should be considered together with the interactions of environmental factors [20].

Nevertheless, relative growth was not good for melons grown in Area 1 according to PCA, which can be related to lower PIES among plants from three different areas. Although the PIES was not directly related to the growth parameters of the melons, the PIES reflects the plant response in relation to complex environmental conditions including the temperature, relative humidity, media water content and CO_2_ concentrations, and the monitoring of the PIES can be used to control growth conditions in smart farms. However, for precise management of smart farm using the PIES, PIES data should be collected for various plants. Time lag between the peaks for PIES and environmental conditions, which was not shown in plants grown in winter, can be different for other plants and should be tested for other plants grown in summer. In addition, to clarify the relationship between the PIES and plant growth parameters, the PIES and plant growth parameters should be investigated under well controlled different environmental conditions.

## Data Availability

The data that support the findings of this study are available from the first author, [J.H. Park], upon reasonable request.
